# Annual Report of the Surgical Clinique of the Jagellonian University, from the 1st of October, 1833, to the 1st of July, 1834

**Published:** 1837-04

**Authors:** 


Art. V.—*Rocznik obejmująncy zdanie sprawy z czynnos'ci Kliniki Chirurgicznéj Uniwersytetu Jagiellon'skiego, od* 1^go^ *Paz'dziernika,* 1833, *do* 1^go^ *Lipca,* 1834, *roku.* Wydał Ludwik Bierkowski, Dyrektor rzeczonego Zakładu, &c. &c.

*Annual Report of the Surgical Clinique of the Jagellonian University, from the* 1*st of October*, 1833, *to the* 1*st of July*, 1834. Published by L. Bierkowski, Director of the said Institution, &c. &c. *Third Year.—Cracow*, 1834. 4to. pp. 37.

The editor of this report is, we presume, the same individual who, in the year 1827, published, in Berlin, a very able and useful work on operative surgery, consisting of a large folio volume of plates, with two thick octavo volumes of description and explanation. Such a proof of industry and professional skill as is therein exhibited would naturally contribute, in no small degree, to the attainment of the honorable situation he held at the time the Report before us was published; while the latter, as a document calculated to throw light on the state of surgical knowledge in Poland, possesses considerable interest, and proves that the unhappy condition of that country has not prevented the extension of medical improvement to its schools: we notice it solely on this account.

The first ten pages contain regulations respecting the exercises to be performed, and qualifications obtained, by medical students of various degrees of standing. Next, after an account of the expenses and some other matters, follows a short description of some of the more important cases treated during the preceding year. The first of these is a case of congenital Fungus hæmatodes, in an infant of six years of age; and, after the extirpation of the tumour, which was between the orbits, the patient died of Encephalitis, and, on examination after death, a communication was discovered between its base and the brain. The rest are, of Elephantiasis; Atresia oris, in consequence of ulceration; Melanosis; and Epulis sarcomatosa. We have then a list of the cases, to the amount of 798, treated in the Stationary and Ambulatory Clinique, corresponding to our hospital and out-practice. Of the above number, 521 were cured, 104 were considerably relieved, 15 were not cured, 8 died, and 12 still remained under treatment. The report closes with a list of the operations performed, operator's names, &c. It is illustrated with five lithographed plates; one illustrating the case of Fungus hæmatodes; two, that of Atresia oris; and the others, cases described in a former Number.

